# The potential role of the HCN1 ion channel and BDNF-mTOR signaling pathways and synaptic transmission in the alleviation of PTSD

**DOI:** 10.1038/s41398-020-0782-1

**Published:** 2020-03-20

**Authors:** Lianghui Ni, Yanling Xu, Sixuan Dong, Yujia Kong, Hong Wang, Guohua Lu, Yanyu Wang, Qi Li, Changjiang Li, Zhongde Du, Hongwei Sun, Lin Sun

**Affiliations:** 1 Department of Clinical medicine, Weifang Medical University, 7166# Baotong West Street, Weifang, Shandong 261053 P. R. China; 2 Department of Psychology, Weifang Medical University, 7166# Baotong West Street, Weifang, Shandong 261053 P. R. China; 3 Department of Psychology, Qingdao University, 16# Qingda First Street, Qingdao, Shandong 266071 P. R. China; 4 School of Public Health and Management, Weifang Medical University, 7166# Baotong West Street, Weifang, Shandong 261053 P. R. China; 5 Department of Neurology, Sunshine Union Hopital, 9000# Yingqian Street, Weifang, Shandong 261000 P. R. China; 6 Department of Psychiatry and Centre for Reproduction Growth and Development, University of Hong Kong, Hong Kong, China

**Keywords:** Depression, Physiology, Molecular neuroscience

## Abstract

The function of the hyperpolarization-activated cyclic nucleotide-gated channel 1 (HCN1) and the expression of brain-derived neurotrophic factor (BDNF) may be involved in the pathogenesis of post-traumatic stress disorder (PTSD). This study aims to explore the role of the HCN1 channel, BDNF, and mTOR in the actions of PTSD and to examine whether synaptic transmission or plasticity is involved in the regulation of this disease. In the present study, rats were exposed to the single prolonged stress and electric foot shock (SPS&S) procedure, which can induce PTSD-like behaviors in rats. ZD7288 was administered by intracerebroventricular (i.c.v.) injection to one experimental group to inhibit the function of the HCN1 ion channel while 8-Br-cAMP was administered to another group to activate the function of the HCN1 ion channel. A series of behavioral tests and biochemical assessments of certain proteins (HCN1, BDNF, and pmTOR) and synaptic ultrastructure in the prefrontal cortex (PFC) and hippocampus (Hip) were then conducted. The SPS&S procedure induced apparent PTSD-like symptoms in rats. The administration of ZD7288 reduced the immobility time and escape latency time in the forced swim test (FST) and water maze test (WMT) with a decreased level of HCN1, upregulated BDNF-mTOR signaling pathways in the PFC and Hip, and synaptic ultrastructure changes in the PFC. In contrast, the administration of 8-Br-cAMP, which led to a higher level of HCN1 in PFC and Hip, resulted in a decreased number of entries to the open arms without significant change in total arm entries in the elevated plus maze test (EPMT) as well as a shorter center square distance and total distance in the open field test (OFT). Extended escape latency time was also observed in the WMT although there was no alteration of BDNF-mTOR signaling pathways and synaptic ultrastructure in the PFC and Hip. Overall, the inhibition of HCN1, which can alleviate PTSD-like behavior of rats by relieving depression and improving learning ability, may be related to the upregulated BDNF-mTOR signaling pathways and synaptic transmission.

## Introduction

Post-traumatic stress disorder (PTSD) is a mental disorder caused by various events that involve threat to the physical integrity of oneself or others. As PTSD is currently understood, its main symptoms are hyperarousal, intrusive memories of the traumatic events, avoidance of reminders of the traumatic events, and emotional numbing^1^. PTSD is also commonly associated with concurrent anxiety and depression symptoms^2^. Further, learning and spatial memory impairments have been often reported in PTSD^2^. PTSD was conceptualized nearly entirely in psychological terms initially, while the biological literature consisted only of sparse psychophysiological observations. Although purely psychological research into PTSD is important, it should be enhanced by research into the neurobiological and chemical mechanisms underlying the disorder for the exploration of the pathogenesis and the treatment target. Recent studies have suggested that several cation channels and signaling pathways may be involved in physiological processes as well as the pathophysiological states of this disease^3,4^. Subsequent neuroendocrinological studies have also revealed changes in hormonal and neuroregulatory factors including cortisol in the hypothalamic–pituitary–adrenal (HPA) axis and oxytocin in PTSD or other stressed animal models^5,6^.

Hyperpolarization-activated cyclic nucleotide-gated (HCN) ion channels, which consist of four subtypes (HCN 1–4), are known to modulate neuronal excitability and activity via the hyperpolarization-activated current, *I*_h_ (also known as h current), composed of sodium and potassium cations. HCN1 appears to be the most predominant isoform expressed in the hippocampus (Hip), prefrontal cortex (PFC), neocortex, and cerebellar cortex. It can be regulated by the nucleotide cyclic adenosine 3',5'-monophosphate (cAMP) and ZD7288^7^. Several studies have pointed out that selective dorsal Hip reduction of HCN1 by short hairpin RNA (shRNA) exhibits reduced behavioral despair in the forced swim test (FST) and anxiolytic effects in the open-field test (OFT) and elevated plus maze test (EPMT)^8^. In our previous studies, we found that the HCN1-related brain-derived neurotropic factor (BDNF) signaling pathways in the PFC are involved in alleviating PTSD-like effects in rats^3^. These findings indicate that HCN1 has a significant effect on the pathogenesis of PTSD, while the definite function of this channel is yet to be known.

BDNF, a kind of neurotrophic factor in the nerve growth factor family, is a critical signaling molecule for nervous system development that continues to play an important role in the maintenance and survival of neurons^9^. There is strong evidence suggesting that BDNF contributes to the pathogenesis of PTSD^10^. In addition, data from animal models and human neuroimaging studies suggest that one of the underlying mechanisms of PTSD might be aberrant synaptic plasticity; since BDNF is recognized as a bolster of synaptic plasticity, its involvement in PTSD seems likely^11^. Recent studies have suggested that both BDNF and another protein-mammalian target of rapamycin (mTOR) have important roles to play in the development of some mental diseases such as depression and anxiety^8,12^. In neurons, mTOR is present at the synaptic region where it modulates the synthesis of locally translated proteins, is upregulated in an activity-dependent manner, and is critical for different forms of synaptic plasticity^13^. mTOR can be influenced by the activities of neuronal surface receptors and channels including NMDA-R and HCN1; mTOR also acts as a node of convergence downstream of these receptors^9,14^. Several signaling pathways and the kinase catalytic domain in the C-terminal end contain the phosphorylation sites that are correlated with higher overall levels of mTOR activity^9,15^. Existing studies suggest the nonnegligible role of the HCN1 channel and BDNF in PTSD; however, the function of underlying molecular mechanisms, including mTOR and cellular processes, remains to be fully evaluated.

This study explores the role of the HCN1 channel, BDNF, and mTOR in the actions of PTSD in order to examine whether synaptic transmission or plasticity is involved in the regulation of this disease. First, the PTSD model was induced in rats by the single prolonged stress and electric foot shock (SPS&S) procedure^16^. Then, ZD7288, a selective HCN blocker, and 8-Br-cAMP, which can increase opening of HCN channels^17^, were injected intracerebroventricularly (i.c.v.) in order to regulate the HCN1 channel in the Hip and PFC. Biochemical tests, including the detection of HCN1, BDNF, and pmTOR (the phosphorylated form of mTOR) proteins, were then performed. The synaptic ultrastructure was examined by transmission electron microscopy (TEM) for further study of the mechanism under HCN1.

## Materials and methods

### Animals

Prior to the experiment, animals were initially screened using a simple field experiment. Rats that were inactive and stressed or anxious were removed by observing the extent of spontaneous activity so as not to affect the experimental results. Forty two male Wistar rats (8-week old) weighing 220–250 g obtained from the Animal Center of Weifang Medical University were used in our study. The rats were allowed a 1-week period to habituate to their housing conditions before the experiment and were housed under controlled environmental conditions (ambient temperature 24 ± 2 °C, humidity 55 ± 15%, 12 h light/12 h dark cycle) with free access to food and water. The experiments were performed in accordance with the National Institutes of Health Guidelines (Use of Laboratory Animals) and were approved by the Animal Care and Use Committee of Weifang Medical University.

### SPS&S procedure

The SPS&S procedures were similar to our previous study^3^. The rats in the PTSD group (*n* = 34) were immobilized for 2 h in the restraint tubes, followed immediately with a 20-min forced swim conducted in a clear acrylic cylindrical tank (46 cm × 20 cm, 24–25 °C water temperature) filled approximately two-thirds with water. After recuperating for 15 min, the rats were anesthetized with ether until loss of consciousness. The animals were then kept in shock cages for a recovery period of 30 min, and one mA current electric shock was applied to the feet via the metal grid for 4 s. The rats then remained in the shock cages for the next 60 s. Two rats died during the SPS&S procedure. After the SPS&S stressors, the rats were left undisturbed in their home cages for 14 days. The control group (*n* = 8) did not undergo any of the processing.

### Testing the rat model of PTSD

After establishing the rat model of PTSD, 8 rats, which were selected from 32 stressed rats according to a random number table, and the control group were tested for PTSD-like symptoms by behavior including assessments of sensitized fear response, anxiety- and depression-like behavior, and learning and memory abilities. All animals went through all behavioral tests following the principle of placing a less stressful test in front to reduce the impact on subsequent tests. The order of the behavioral testing was (1) freezing behavior test; (2) open field test; (3) elevated plus maze test; (4) water maze test; (5) forced swimming test. Biochemistry assays were taken, including tests of the plasma cortisol and oxytocin levels (*n* = 8), the expression of HCN1, BDNF, and pmTOR, and the synaptic ultrastructure (*n* = 3).

### Experimental group design and drug delivery

After the model testing, the rest of the 24 rats in the PTSD group were randomly assigned into three groups as follows: PTSD + Vehicle (*n* = 8), PTSD + ZD7288 (*n* = 8), or PTSD + 8-Br-cAMP (*n* = 8). Drugs were administered by i.c.v. injection. ZD7288 (a single dose of 5 µg) and 8-Br-cAMP (a single dose of 100 µg) were dissolved in normal saline. The drugs were delivered into the right or left lateral ventricle (0.8 mm posterior to bregma, 1.5 mm lateral to midline, 3.5 mm ventral to dura) at the rate of 0.5 µl/min via a 5 µl Hamilton syringe. Following at least 5 days of recovery, the rats were tested for behavior^17^.

### Behavior tests

The operators made minimal movements and no noise when performing the experiment and collecting behavioral data. All observation and recording for animal ethology was used by an automated animal behavior observation system (SMART, Panlab SL, Barcelona, Spain). Experimenters were blind to experimental group at the time of the experiment. Analysis was also blind to different group, as it was done by automatic software. (Further, in the large testing rooms, we hung a curtain from the ceiling that separates the area where the apparatus and operators are located, minimizing the possibility of experimental animals reacting to the operator’s presence.) All animals went through all behavioral tests, which were conducted in the same sequence: (1) open field test; (2) elevated plus maze test; (3) water maze test; (4) forced swimming test.

### Open field test (OFT)

The OFT is a behavioral test for assessing exploration activity and anxiety level in rats^18,19^. An enclosed square box made of dark opaque Plexiglas (100 cm × 100 cm × 50 cm) was used. The field was divided into 25 squares using virtual grid lines for analysis. Rats were individually and gently placed in the middle area of the open field facing the same direction for each trial. Excrement was cleaned, and the apparatus was scrubbed with 70% ethanol between each test to eliminate any residual olfactory cues from the previously tested rat. The distance that rats traveled within 5 min in the center square as well as the total distance were recorded.

### Elevated plus maze test (EPMT)

The EPMT has been described as a simple method for assessing anxiety responses and exploration ability of rodents^20^. The maze is made of stainless steel painted black and consists of four arms (two open without walls and two enclosed by 40 cm high walls) 50 cm long and 10 cm wide with a center platform (10 cm × 10 cm). The apparatus was positioned at 100 cm above the floor. Each rat was gently placed on the central platform with its head oriented to the closed arm, and they were allowed to explore the maze for 5 min freely. The entries of open arms, closed arms, the center area of the maze, and the time spent in each of these areas were detected and recorded. The excrement was also cleaned, and the apparatus was scrubbed with 70% ethanol between each test.

### Freezing behavior test (FBT)

To test for sensitized fear response (hyperarousal), 24 h after finishing the SPS&S procedure rats were placed into the neutral test chamber for 3 min. A neutral tone (80 dB, 9 kHz) was presented for 3 min then. After the tone presentation, they remained in the test chamber for another 60 s before being returned to the home cages. The freezing behavior of the animals was recorded and analyzed later. Freezing was defined as the lack of all observable movement of any parts of the body and vibrissae; except for those movements related to respiration, all other behavior was scored as active^16^.

### Water maze test (WMT)

The WMT is used to measure spatial navigation learning and memory in rats. Spatial learning is assessed across repeated trials (place navigation test), and preference for the platform area when the platform is absent (spatial probe test) determines the reference memory^21^. The apparatus is a black circular tank (150 cm in diameter) filled with water (25 cm, 23 ± 1 °C). Four signs (square, heart-shape, moon, and triangle) were placed around the tank at four fixed points in each quadrant, and the platform was hidden 1 cm below the water surface in the center of one quadrant of the pool. The place navigation test was conducted during the first four days with four swim trials given per day, in which each animal was released from a different quadrant. A maximum of 90 s was allowed for each trial; if the rat did not find the platform within 90 s, it was guided to the platform and allowed to remain there for 10 s. The latency to escape onto the platform was recorded. In the spatial probe test, the platform was removed, and rats were placed at a quadrant randomly to explore at liberty for 90 s. In this session, the percentage of time in the target quadrant where the platform was initially placed was recorded.

### Forced swim test (FST)

Depressive-like behavior of rats was tested by the FST^22^. Rats were individually forced to swim in a cylindrical glass tank (46 cm × 20 cm in diameter) containing 36 cm of water (24−25 °C). Two swimming sessions were performed with an initial 15-min ‘pre-test’ followed by a 5-min ‘test’ after 24 h. In the 5-min test, we recorded passive behavior (immobility time), which is defined as time during which the animal floats in the water, making only those movements necessary to keep its head above the water.

### Biochemical tests

#### Enzyme-linked immune sorbent assay (ELISA)

Plasma cortisol and oxytocin levels were determined by ELISA. On D15, the rats for model testing were sacrificed and their blood was taken. On D22, all remaining rats were sacrificed; their blood was taken for ELISA at that time. The number of rats used from each group was SPS&S *n* = 8; control *n* = 8; SPS&S + Vehicle *n* = 4; SPS&S + ZD7288 *n* = 4; SPS&S + 8-Br-cAMP *n* = 4. Blood was collected into iced heparinized tubes. The collected blood samples were centrifuged at 3000 rpm for 20 min at 4 °C. Two milliliters of plasma was removed and collected into eppendorf tubes. Cortisol and oxytocin levels were determined using a cortisol ELISA kit (AD3213Mo, Andygene, Beijing) and an oxytocin ELISA kit (AD3213Mo, Andygene, Beijing), respectively. The absorbance of the reference standard and specimen were detected under a wavelength of 450 nm after incubation, washing, and coloration according to the ELISA kit directions (AD3213Mo and AD3031Mo, Andygene, Beijing).

#### Western blot assay

To analyze the protein expression of HCN1, BDNF, and pmTOR, the number of animals used from each group was SPS&S *n* = 4; control *n* = 4; SPS&S + Vehicle *n* = 4; SPS&S + ZD7288 *n* = 4; SPS&S + 8-Br-cAMP *n* = 4. Western blot assay was conducted as we previously published^3^. Rats were decapitated, and the brains were immediately removed. The PFC and Hip were dissected out, placed in liquid nitrogen, and stored at −80 °C until used. The assay was performed with protein extracted using radioimmunoprecipitation assay buffer (R0010, Solarbio, China), and the protein concentration was determined by the bicinchoninic acid assay. The protein was separated on 8–15% sodium dodecyl sulfate-polyacrylamide gel and transferred to a polyvinylidene fluoride membrane. The membrane was incubated with blocking solution (5% dried milk powder in phosphate-buffered saline, 0.05% Tween 20, pH 7.4) (PBST) for 2 h. The membrane was incubated in primary antibody overnight at 4 °C, then rinsed in PBST buffer for 20 min, followed by being incubated in secondary antibody for 1 h at room temperature. It was combined with the Immobilon Western Chemiluminescent HRP Substrate (WBKLS0050, Millipore, Billerica, MA, USA) for color reaction. Primary antibodies used were as follows: Anti-HCN1 (1:200, ab84816, Abcam, England), Anti-BDNF (1:500, ab205067, Abcam, England), and Anti-pmTOR (1:200, #5536, Cell Signaling Technology, USA). The gray value of immune reactivity was quantified using Image-Pro Plus (6.0).

#### Immunofluorescence assay

Three rats were used from each group. The immunofluorescence assay was carried out similarly to previous works^23,24^. The rats were deeply anesthetized and perfused transcardially with 4% paraformaldehyde. Brains were removed and immersed in the same fixative overnight at 4 °C, followed by dehydration and paraffin imbedding. Coronal sections (60 μm) were cut using a cryostat. After rehydration, the sections were blocked for 1 h at 37 °C in PBS containing 5% goat serum and 0.3% TritonX-100 and were incubated alone with primary antibody. After being washed again in PBS, sections were further incubated in secondary antibody, and the nucleus was labeled with Hoechst (Hoechst 33342, Solarbio, China). Finally, the sections were rinsed in PBS and covered with glass slides. All immunofluorescence-labeled sections were viewed with a fluorescence microscope (Olympus Corporation, Japan). Primary antibodies used were as follows: Anti-HCN1 (1:200, ab84816, Abcam, England), Anti-BDNF (1:3000, ab205067, Abcam, England), and Anti-pmTOR (1:200, #5536, Cell Signaling Technology, USA). Secondary antibodies used were Alexa Fluor 594: conjugated Goat Anti-Mouse IgG (SA00006-3, Proteintech, USA) and Alexa Fluor 488: conjugated AffinipureGoat Anti-Rabbit IgG (SA00006-2, Proteintech, USA).

#### Transmission electron microscopy (TEM)

We randomly sampled four rats to detect the variation in synaptic ultrastructure. The TEM test was carried out to analyze the ultrastructure of synapses^25^. The rats was anaesthetized and perfused through the ascending aorta initially with 0.9% NaCl in 0.01 M sodium-potassium phosphate buffer pH 7.4 and after with 2% paraformaldehyde and 2.5% glutaraldehyde in 0.1 M cacodylate buffer, pH 7.4 at 20 °C (Sigma-Aldrich, Poland). Material for ultrastructural studies was sampled from the Hip and PFC. Specimens were fixed in ice-cold fixative solution for 20 h and placed in a mixture of 1% OsO_4_ and 0.8% K4[Fe(CN)6]. After dehydration in a series of ethanol gradients, tissue specimens were embedded in epoxy resin (Agar Scientific, Essex, UK). Ultra-thin sections (60 nm) were examined by TEM (JEM-1200EX, Jeol, Japan). The ultrastructural changes in PFC and Hip synapses were observed under TEM (HT7700-SS, Hitachi, Tokyo, Japan). Postsynaptic density (PSD), synaptic cleft width, and curvature of the synaptic interface were quantified using Image-Pro Plus (6.0) as described by Jones and Guldner^26,27^.

### Statistical analysis

We estimated the sample size by software PASS.11 (NCSS, USA). And pilot experiment was carried out to determine effect sizes according to the preliminary experimental results. All data were expressed as mean ± SEM and analyzed by SPSS statistical software (Version 22.0, SPSS, Inc.; Chicago, IL, USA). The normality and variance of data distribution between two groups were analyzed by Kolmogorov–Smirnov test and Levene’s test, respectively (*P* > 0.05). The data between the control and SPS&S groups were analyzed by Student’s *t*-test. The one-way analysis of variance (ANOVA) was used for the analysis of the behavioral results of the OFT, EPMT, FST, probe test in WMT, and chemistry results followed by a post-hoc test (Dunnett’s test) in the PTSD + Vehicle, PTSD + ZD7288, and PTSD + 8-Br-cAMP groups. A repeated measures multivariate analysis of variance (MANOVA) was used for the analysis of escape latency time in WMT. The relationship between the level of BDNF and pmTOR was described by linear regression, and the effect of BDNF between HCN1 and pmTOR was measured by the mediator effect test. *p* < 0.05 was considered as statistically significant.

## Results

### Effects of SPS&S procedure

**Fig. 1:**
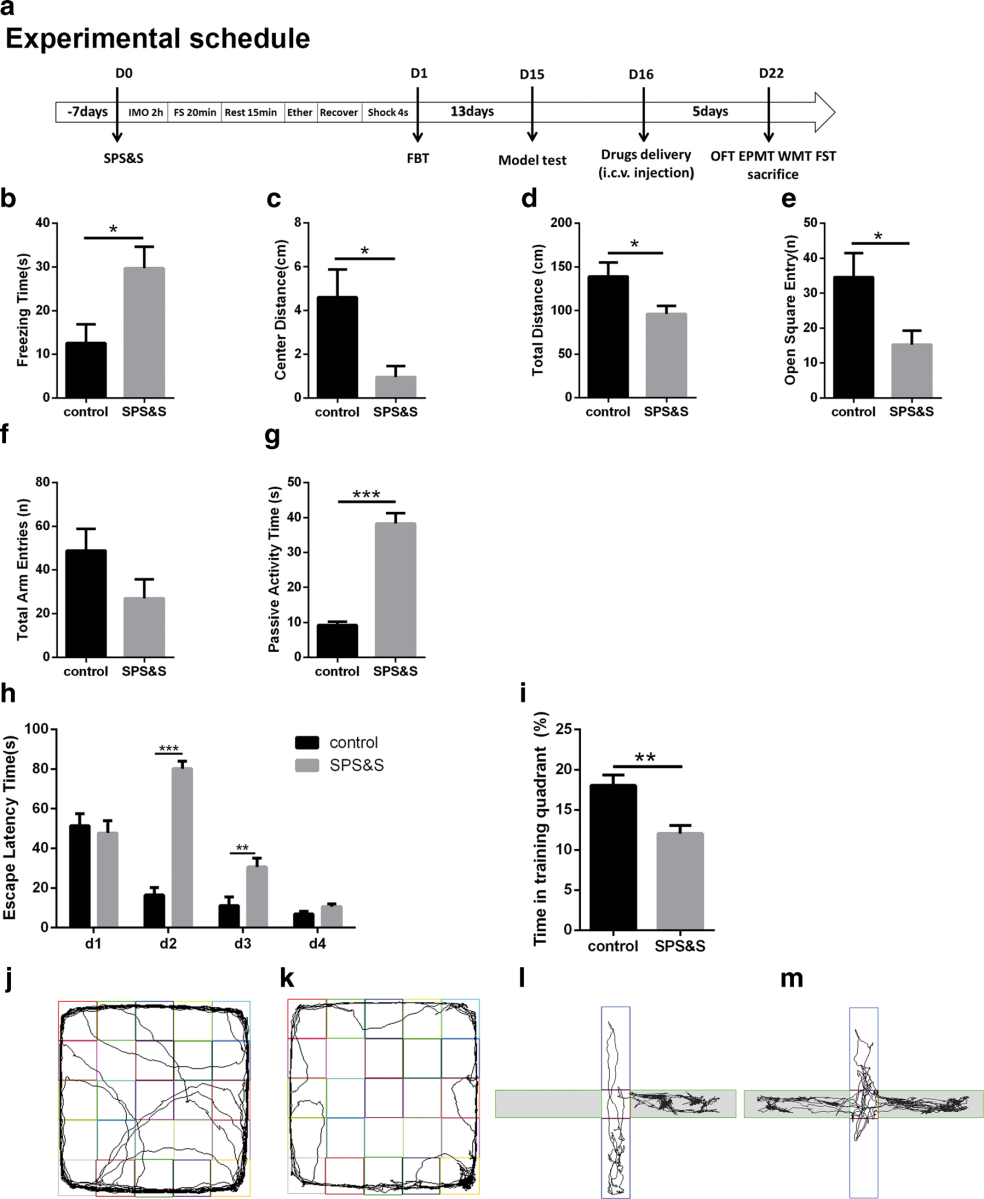
Experimental schedule and effects of SPS&S procedure on behavior test. **a** After a 1-week period to habituate to their housing conditions, the rats in the PTSD group rats were exposed to single prolonged stress and electric foot shock (SPS&S), including being immobilized for 2 h (IMO 2 h), forced swim for 20 min (FS 20 min), rest period for 15 min (Rest 15 min), exposure to ether until loss of consciousness (Ether), followed by a 30 min recovery. The rats were then shocked for 4 s (Shock 4 s). On the second day after SPS&S, the rats were tested for freezing behavior (FBT). After an undisturbed period in their home cages, the model test was conducted to rats, and the drugs were administrated via intracerebroventricular (i.c.v.) injection. Behavior tests were assessed after 5 days recovery including the open field test (OFT), the elevated plus maze test (EPMT), the water maze test (WMT) and the forced swim test (FST). **b** The freezing time in FBT (Student’s *t*-test: *t* = −2.643, *p* = 0.019). **c**, **d** The distance traveled in the center square (Student’s *t*-test: *t* = 2.650, *p* = 0.026) and total distance (*t* = 2.354, *p* = 0.038) in OFT. **e**, **f** The entries in the open arms (Student’s *t*-test: *t* = 2.436, *p* = 0.029) and total arm entries (*t* = 1.646, *p* = 0.122) in EPMT. **g** The passive activity time in FST (Student’s *t*-test: *t* = −9.130, *p* = 0.000). **h**, **i** The escape latency time in place navigation test (MANOVA: *F*(3, 42) = 27.992, *p* = 0.000; the second day: *p* = 0.000 and third day: *p* = 0.007) and time spend in training quadrant in spatial probe test (Student’s *t*-test: *t* = 3.626, *p* = 0.003) of WMT. **j**, **k**, **l**, **m** Representative video tracking images during EMPT and OFT, **j**, **l** control group, **k**, **m** SPS&S group. Data are represented as mean ± SEM. *p < 0.05 was expressed statistically significant. *p < 0.05, **p < 0.01, and ***p < 0.001 compared with control group.

**Fig. 2:**
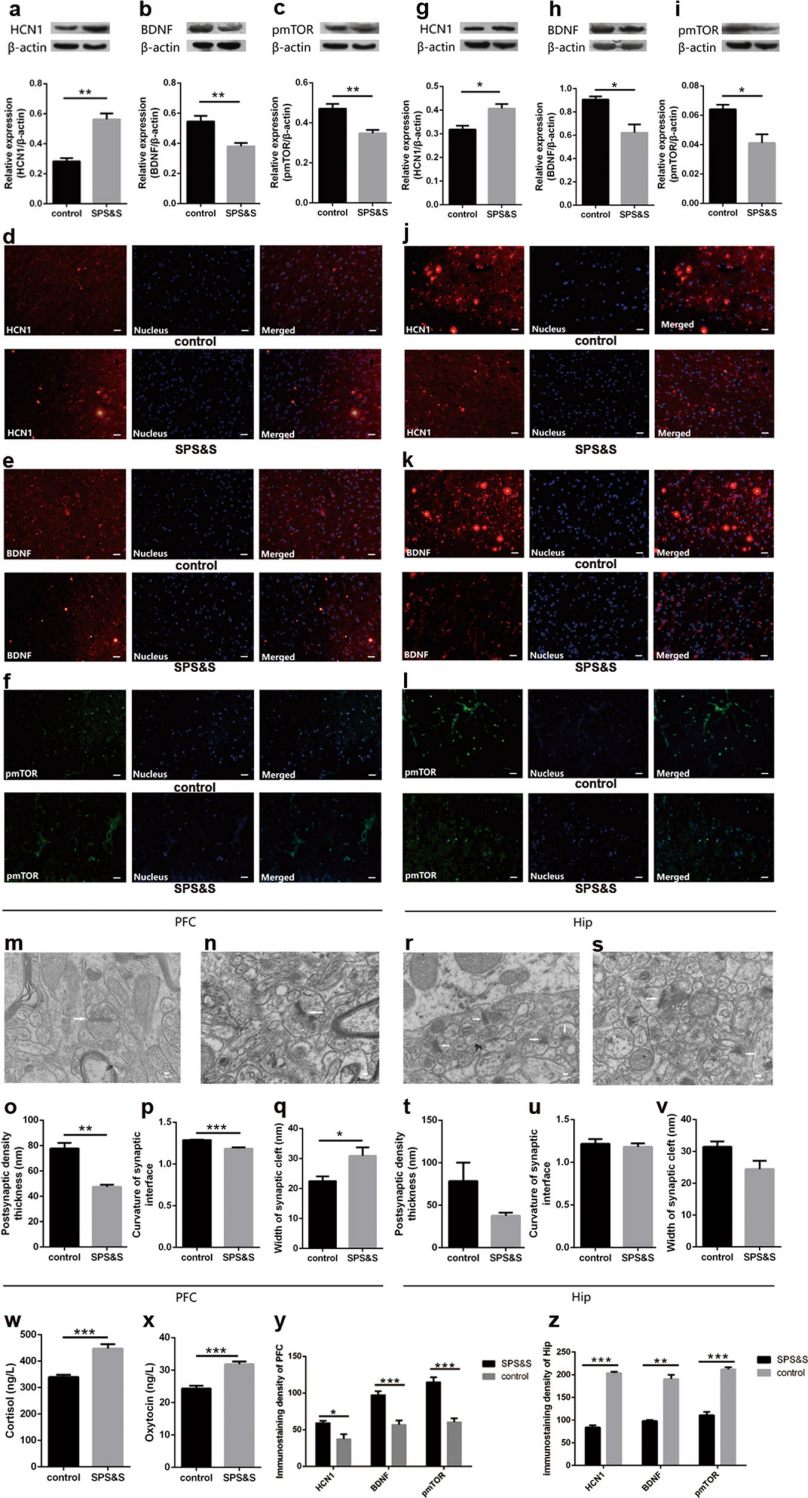
Effects of SPS&S procedure on relative protein expression normalized by β-actin and synaptic ultrastructure in the PFC and Hip and levels of cortisol and oxytocin in blood. **a**–**c** The expression of HCN1 (Student’s *t*-test: *t* = −6.255, *p* = 0.001), BDNF (*t* = 3.805, *p* = 0.009) and pmTOR (*t* = 4.226, *p* = 0.006) in PFC. **g**–**i** The expression of HCN1(Student’s *t*-test: *t* = −3.579, *p* = 0.012), BDNF (*t* = 3.733, *p* = 0.022) and pmTOR (*t* = 3.424, *p* = 0.014) in Hip. **d**–**f**, **j**–**l** Localization and distribution of HCN1, BDNF and pmTOR in PFC (**d**–**f**) and (**j**–**l**). Bar = 20 μm. **o**–**q** The PSD (Student’s *t*-test: *t* = 6.499, *p* = 0.001), curvature of synaptic interface (*t* = 7.021, *p* = 0.003) and width of synaptic cleft (*t* = −2.628, *p* = 0.039) in the PFC. **t**–**v** The PSD (Student’s *t*-test: *t* = 1.846, *p* = 0.158), curvature of synaptic interface (*t* = 0.499, *p* = 0.635) and width of synaptic cleft (*t* = 2.257, *p* = 0.065) in the Hip. **m**, **n**, **r**, **s** Synaptic ultrastructure in PFC (**m**, **n**) and Hip (**r**, **s**) under ×10,000 magnification. **w**, **x** The level of cortisol (Student’s *t*-test: *t* = −5.694, *p* = 0.000) and oxytocin (*t* = −6.508, *p* = 0.000) in blood. **y** Immunoreactivity analysis of HCN1 (Student’s *t*-test: *t* = 3.147, *p* = 0.0346), BDNF (*t* = 5.227, *p* = 0.006), and pmTOR (*t* = 6.184, *p* = 0.003) in PFC. **z** Immunoreactivity analysis of HCN1 (Student’s *t*-test: *t* = 21.82, *p* = 0.000), BDNF (*t* = 9.956, *p* = 0.001), and pmTOR (*t* = 11.23, *p* = 0.000) in Hip. Data are represented as mean ± SEM. *p < 0.05 was expressed statistically significant. *p < 0.05, **p < 0.01, and ***p < 0.001 compared with control group.

Student’s *t*-test was used to analyze SPS&S procedure effects between the SPS&S group and control group except for the data from the place navigation test in WMT. Rats in the SPS&S group showed more freezing time in FBT (Fig. 1b) as a consequence of the hyperarousal state. They also displayed decreased exploration activity presented as decreased total distance in OFT (Fig. 1d), reduced distance traveled in the center square (Fig. 1c) in OFT, and obvious anxiety-like behaviors as defined by the fewer number of entries in the open arms without significant change in total arm entries in EPMT (Fig. 1e, f). Compared with the control group, the SPS&S group also displayed significantly increased duration of passive activity in FST (Fig. 1g), implying depression-like behavior. For spatial learning memory, the data of the WMT showed that the interaction was statistically significant after the SPS&S procedure was analyzed by MANOVA. The SPS&S group had an increased escape latency time on the second and third test days in the place navigation test (Fig. 1h) and decreased time in the training quadrant in the spatial probe test (Fig. 1i). From the results of the Western blot and immunofluorescence assay (Fig. 2a–l), the expression of HCN1, BDNF, and pmTOR in the PFC and Hip were lower in the SPS&S group than those in the control group. The results of TEM (Fig. 2m–v) showed that the postsynaptic density thickness and curvature of synaptic interface were reduced, and the width of synaptic cleft was augmented in the PFC of the SPS&S group compared to those of control group; differences were not detected in the Hip. The detection of plasma showed that cortisol (Fig. 2w) and oxytocin (Fig. 2x) rose significantly in the SPS&S group*.*

### Effects of ZD7288 and 8-Br-cAMP on PTSD-like behaviors under the SPS&S procedure

**Fig. 3:**
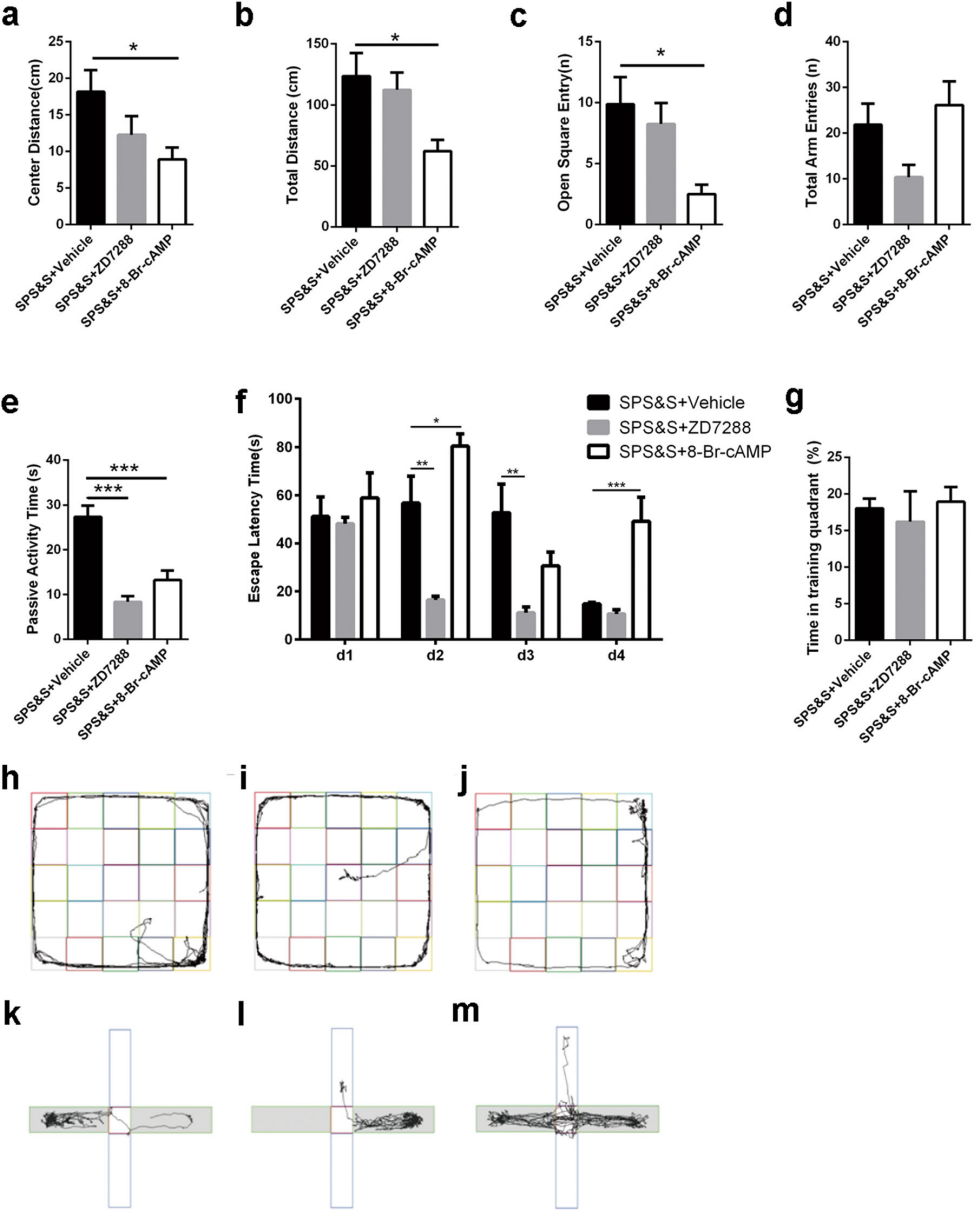
Effects of ZD7288 and 8-Br-cAMP on PTSD-like behaviors under SPS&S procedure. **a**, **b** The distance traveled in the center square (one-way ANOVA: *F*(2, 21) = 3.668, *p* = 0.043; Post-hoc test: SPS&S + ZD7288: *p* = 0.179, SPS&S + 8-Br-cAMP: *p* = 0.026) and total distance (one-way ANOVA: *F*(2, 21) = 4.924, *p* = 0.018; Post-hoc test: SPS&S + ZD7288: *p* = 0.811, SPS&S + 8-Br-cAMP: *p* = 0.014) in OFT. **c**, **d** The entries in the open arms (one-way ANOVA: *F*(2, 21) = 5.340, *p* = 0.013; Post-hoc test: SPS&S + ZD7288: *p* = 0.720, SPS&S + 8-Br-cAMP: *p* = 0.010) and total arm entries (one-way ANOVA: *F*(2, 21) = 3.592, *p* = 0.046; Post-hoc test: SPS&S + ZD7288: *p* = 0.128, SPS&S + 8-Br-cAMP: *p* = 0.711) in EPMT. **e** The passive activity time in FST (one-way ANOVA: *F*(2, 21) = 23.944, *p* = 0.000; Post-hoc test: SPS&S + ZD7288: *p* = 0.000, SPS&S + 8-Br-cAMP: *p* = 0.000). **f**, **g** The escape latency time in place navigation test (MANOVA: *F*(6, 63) = 5.902, *p* = 0.000; SPS&S + ZD7288:the second day: *p* = 0.001 and third day: *p* = 0.001; SPS&S + 8-Br-cAMP: the second day: *p* = 0.030 and fourth day: *p* = 0.000)and time spend in training quadrant in spatial probe test (one-way ANOVA: *F*(2, 21) = 0.251, *p* = 0.781; Post-hoc test: SPS&S + ZD7288: *p* = 0.855, SPS&S + 8-Br-cAMP: *p* = 0.963) of WMT. **h**–**m** Representative video tracking images during EMPT and OFT, **h**, **k** SPS&S + Vehicle group, **i**, **l** SPS&S + ZD7288 group, **j**, **m** SPS&S + 8-Br-cAMP group. Data are represented as mean ± SEM. *p < 0.05 was expressed statistically significant. *p < 0.05, **p < 0.01, and ***p < 0.001 compared with SPS&S + Vehicle group.

The SPS&S + 8-Br-cAMP group had a decreased number of entries to the open arms without a significant change in total arm entries in the EPMT (Fig. 3c, d). This as well as the shorter distances traveled in the center square (Fig. 3a) and in total distance (Fig. 3b) in the OFT demonstrate that the activation of HCN1 can lead to severe anxiety-like behavior and reduced exploration ability compared with the SPS&S + Vehicle group. Results showed a remarkably decreased duration of passivity of rats during the forced swim; decreased duration of passivity was also found in the SPS&S + 8-Br-cAMP group (Fig. 3e). The analysis of the place navigation test in WMT (Fig. 3f) showed that on the second and third test days, the escape latency time of the SPS&S + ZD7288 group decreased*.* The SPS&S + 8-Br-cAMP group had a longer escape latency time on the second and fourth test days.

### Effects of ZD7288 and 8-Br-cAMP on the expression of HCN1, BDNF, and pmTOR in the PFC and Hip under the SPS&S procedure

**Fig. 4:**
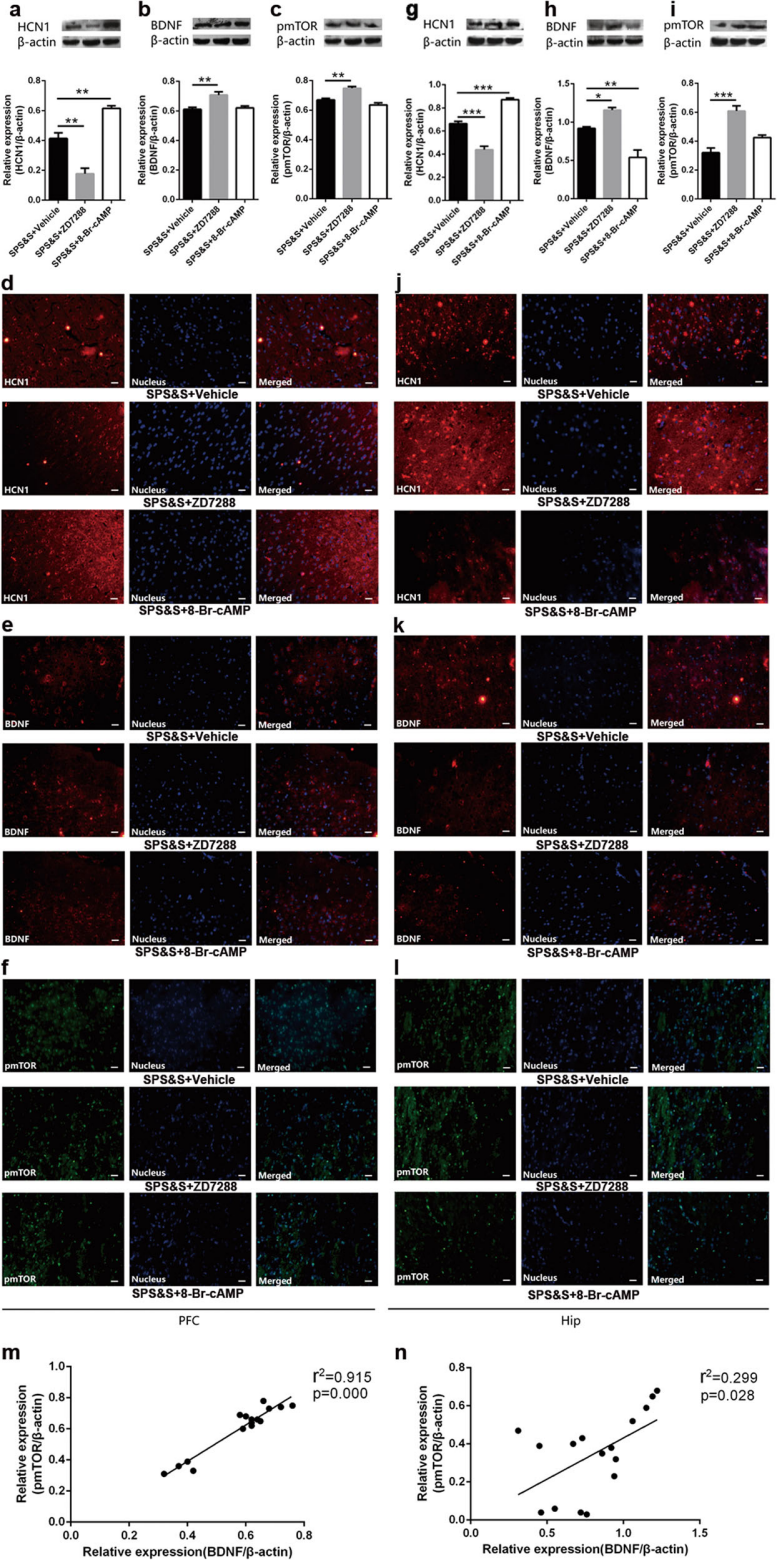
Effects of ZD7288 and 8-Br-cAMP on the expression of relative protein in the PFC and Hip under SPS&S procedure normalized by β-actin. **a**–**c** The expression of HCN1 (one-way ANOVA: *F*(2, 9) = 46.185, *p* = 0.000; Post-hoc test: SPS&S + ZD7288: *p* = 0.001, SPS&S + 8-Br-cAMP: *p* = 0.003), BDNF (one-way ANOVA: *F*(2, 9) = 10.446, *p* = 0.005; Post-hoc test: SPS&S + ZD7288: *p* = 0.005, SPS&S + 8-Br-cAMP: *p* = 0.885) and pmTOR (one-way ANOVA: *F*(2, 9) = 23.233, *p* = 0.000; Post-hoc test: SPS&S + ZD7288: *p* = 0.002, SPS&S + 8-Br-cAMP: *p* = 0.135) in PFC. **g**–**i** The expression of HCN1 (one-way ANOVA: *F*(2, 9) = 91.914, *p* = 0.000; Post-hoc test: SPS&S + ZD7288: *p* = 0.000, SPS&S + 8-Br-cAMP: *p* = 0.000), BDNF(one-way ANOVA: *F*(2, 9) = 26.364, *p* = 0.000; Post-hoc test: SPS&S + ZD7288: *p* = 0.039, SPS&S + 8-Br-cAMP: *p* = 0.003) and pmTOR(one-way ANOVA: *F*(2, 9) = 24.342, *p* = 0.000; Post-hoc test: SPS&S + ZD7288: *p* = 0.000, SPS&S + 8-Br-cAMP: *p* = 0.063) in Hip. **d**–**f**, **j**–**l** Localization and distribution of HCN1, BDNF and pmTOR in PFC (**d**–**f**) and (**j**–**l**). Bar = 20 μm. **m**, **n** The correlation between the level of BDNF and pmTOR in the PFC (**m**) and Hip (**n**) analyzed with linear regression. Data are represented as mean ± SEM. *p < 0.05 was expressed statistically significant. *p < 0.05, **p < 0.01, and ***p < 0.001 compared with SPS&S + Vehicle group. The mediator effect test of BDNF (bootstrap) with a sample size of 5000 and 95% confidence interval showed that the BDNF was a mediator between HCN1 and pmTOR in the PFC (model: X_pmTOR_ = 0.0104×_HCN1_ + 0.9152×_BDNF_, confidence interval: [−0.7259, −0.0211], effect: −0.3802) and Hip (model: X_pmTOR_ = 0.7760×_HCN1_ + 0.7211×_BDNF_, confidence interval: [0.4280,1.1241], effect: 0.3186).

The rats treated with ZD7288 and 8-Br-cAMP illustrated variations in the expression of HCN1, BDNF, and pmTOR in the PFC and Hip (Fig. 4a–l). The results of the Western blot assay showed that there was a decreased level of HCN1 and higher expressions of BDNF and pmTOR in the rats treated with ZD7288 in PFC and Hip compared with the SPS&S + Vehicle group. The level of HCN1 in the SPS&S + 8-Br-cAMP group was higher in the PFC and Hip than that in the SPS&S + Vehicle group while a lower expression of BDNF was detected in the Hip with no difference in the PFC and no variation of pmTOR in the PFC or Hip. Further correlation analysis indicated a positive correlation between the level of BDNF and pmTOR in the PFC (Fig. 4m) and Hip (Fig. 4n), and the mediator effect test showed that BDNF was a mediator between HCN1 and pmTOR.

### Effects of ZD7288 and 8-Br-cAMP on the synaptic ultrastructure in the PFC and Hip under the SPS&S procedure

**Fig. 5:**
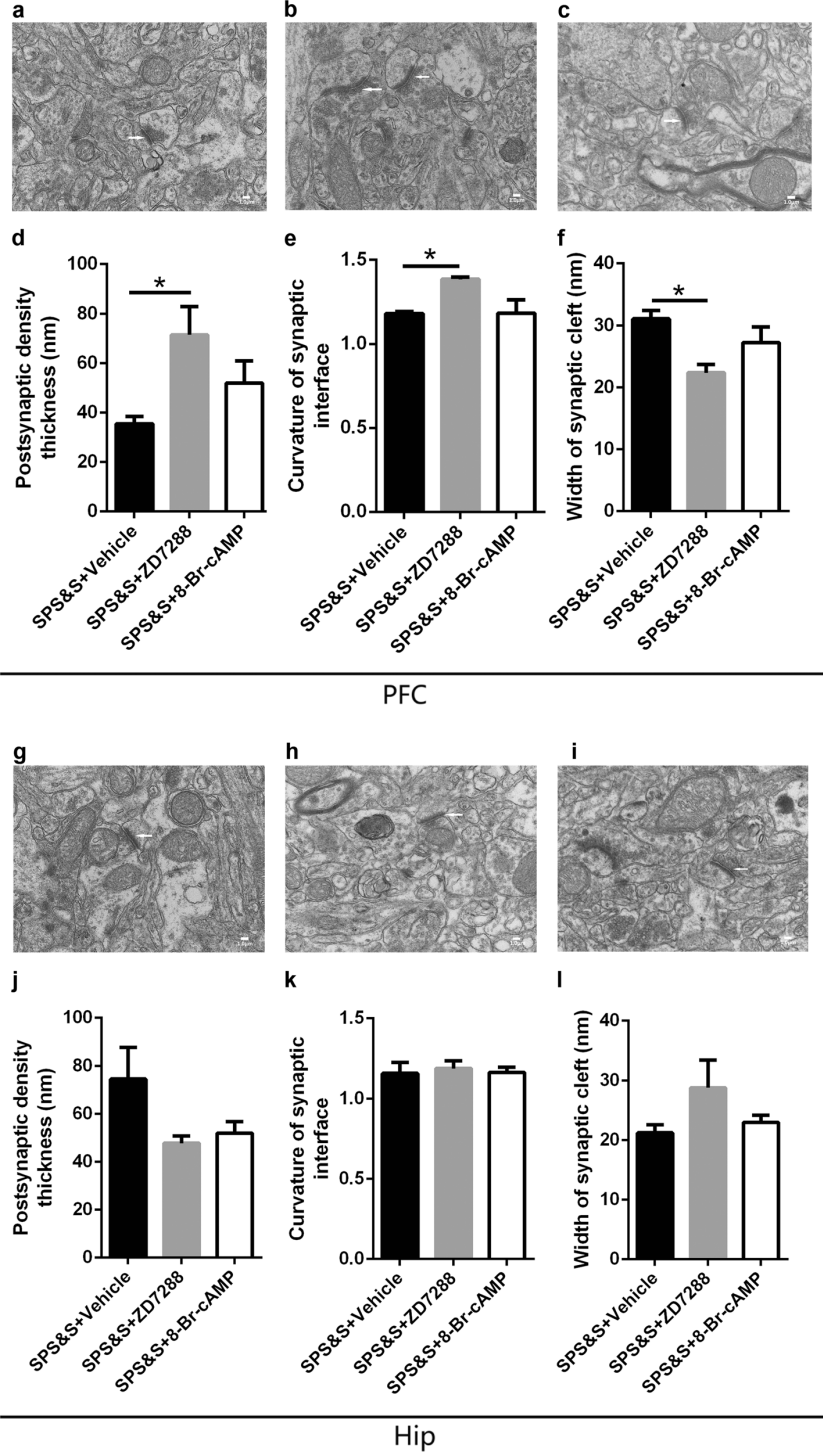
Effects of ZD7288 and 8-Br-cAMP on the synaptic ultrastructure in the PFC and Hip under SPS&S procedure. **a**–**c** The PSD (one-way ANOVA: *F*(2, 9) = 4.416, *p* = 0.046; Post-hoc test: SPS&S + ZD7288: *p* = 0.028, SPS&S + 8-Br-cAMP: *p* = 0.338), curvature of synaptic interface (one-way ANOVA: *F*(2, 9) = 6.005, *p* = 0.022; Post-hoc test: SPS&S + ZD7288: *p* = 0.026, SPS&S + 8-Br-cAMP: *p* = 0.999) and width of synaptic cleft (one-way ANOVA: *F*(2, 9) = 5.836, *p* = 0.024; Post-hoc test: SPS&S + ZD7288: *p* = 0.014, SPS&S + 8-Br-cAMP: *p* = 0.277) in the PFC. **g**–**i** The PSD (one-way ANOVA: *F*(2, 9) = 3.002, *p* = 0.100; Post-hoc test: SPS&S + ZD7288: *p* = 0.086, SPS&S + 8-Br-cAMP: *p* = 0.148), curvature of synaptic interface (one-way ANOVA: *F*(2, 9) = 0.109, *p* = 0.898; Post-hoc test: SPS&S + ZD7288: *p* = 0.863, SPS&S + 8-Br-cAMP: *p* = 0.986) and width of synaptic cleft (one-way ANOVA: *F*(2, 9) = 1.901, *p* = 0.205; Post-hoc test: SPS&S + ZD7288: *p* = 0.162, SPS&S + 8-Br-cAMP: *p* = 0.872) in the Hip. **d**–**f**, **j**–**l** Synaptic ultrastructure in PFC (**d**–**f**) and Hip (**j**–**l**) under ×10,000 magnification. Data are represented as mean ± SEM. *p < 0.05 was expressed statistically significant. *p < 0.05, **p < 0.01, and ***p < 0.001 compared with control group.

In the PFC, the PSD and the width of the synaptic cleft were changed in synaptic ultrastructure. A one-way ANOVA among the SPS&S + Vehicle, SPS&S + ZD7288, and SPS&S + 8-Br-cAMP groups indicated that, after delivery of ZD7288, there was increased PSD (Fig. 5d), curvature of the synaptic interface (Fig. 5e), and more narrow width of the synaptic cleft (Fig. 5f). However, the changes were not detected in the Hip, and the administration of 8-Br-cAMP had no effect on the synaptic ultrastructure in the PFC or Hip.

## Discussion

PTSD is a debilitating disorder associated with functional impairments, physical health concerns, and mental health comorbidities. Exposure to severe life-threatening or traumatic events can increase the emergent risk of PTSD symptoms^28,29^. However, the treatment of PTSD is extremely challenging and may include many years of individual and group therapy and medications. Therefore, the establishment of an appropriate animal model of PTSD is crucial to promoting our understanding of PTSD. Further, an animal model of PTSD and a better understanding of its mechanisms may help to identify novel and more effective therapeutic strategies.

Among the various methods proposed to establish animal models of PTSD, the SPS&S procedure was used in our experiment. This modified model of classic SPS is a valid animal model that can replicate the specific neuroendocrinological and behavioral abnormalities of PTSD—for example, excessively responsive HPA system^6^, enhanced fear responses, and anxiety-and depression-like behaviors^16^. In contrast, other models, such as the pre-shock stress model and predator stress model, cannot mimic pathophysiological abnormalities^30,31^. There are three validation criteria typically used to assess the validity of an animal model: phenomenological similarity (face validity), corresponding theoretical explanatory frameworks (construct validity), and the ability to predict whether a pharmacological agent with efficacy demonstrated in animal studies has a subsequent therapeutic effect in humans (predictive validity)^32^. The plentiful research supporting the face and construct validity of the SPS&S model suggests that it could be suitable for the study of PTSD^3,32^. With respect to face validity, the results of model testing showed that SPS&S rats exhibit symptoms of increased arousal such as exaggerated sensitized fear responses, decreased exploration activity, increased anxiety-and depression-like behaviors, and impaired learning and memory. It is worth mentioning that the OFT and EPMT both can detect the degree of anxiety; the OFT is suited to measuring locomotion activity, whereas the EPMT is suited to explore animals’ reactions to novelty in new and different environments. Moreover, forced swimming test has two roles in this study. In SPS&S we used forced swimming as a psychological stressor and were used in behavioral tests to assess depression levels in rats. Regarding construct validity, the higher expression of HCN1 and decreased level of BDNF and pmTOR signaling were examined both in the PFC and Hip. Our studies also suggest a synaptic transmission abnormality in the PFC. Moreover, the rats’ increased release of cortisol and oxytocin in the blood imply a response to stressors. Although pharmacotherapy for PTSD is still at a relatively inchoate phase of development, early intervention with paroxetine during the incubation period has proven to be effective in ameliorating SPS&S-induced PTSD symptoms^16^.

Past studies have shown evidence that HCN1 was expressed higher and the BDNF level was lower in the PFC in rat models of PTSD; several studies of PTSD patients also found dysfunctional BDNF^3,33–37^. In line with these findings, the SPS&S procedure in our study increased the level of HCN1, which led to a lower expression of BDNF, not only in the PFC but also in the Hip. These regions in the limbic system are important for neural structures and emotional processing in humans and animals; these regions have been reported to clearly alter in PTSD^11,38^. By analyzing the results of TEM we found that rats in the SPS&S group had decreased thickness of PSD, curvature of the synaptic interface, and a wider synaptic cleft in the PFC. PSD is involved in the postsynaptic membrane response to the neuron signal while the curvature of the synaptic interface is related to the contact area between neurons. The synaptic cleft width may be associated with the delivery of neurotransmitters from the presynaptic membrane to the postsynaptic membrane. All of these changes could reflect the transmission efficiency of neuron system^25,39^. In fact, it has been found that the pathophysiology of PTSD can be linked to several neurobiological mechanisms related to synaptic plasticity that can alter the behavior of neural circuits by modifying the strength or efficacy of synaptic transmission;^11^ thus, our findings indicate that the SPS&S procedure resulted in a synaptic transmission, even synaptic plasticity deficit, in rats. If the SPS&S procedure affects synaptic plasticity in rats similarly to how PTSD affects synaptic plasticity in humans, then the SPS&S procedure can be said to have predictive validity. It can therefore be confirmed that the SPS&S procedure can mimic the clinical characteristics of PTSD with face, construct, and predictive validity in the present study.

The changes of HCN1, BDNF, and synaptic ultrastructure in the PTSD animal model of rats have been detected. To further determine whether HCN1 has an influence on some cellular signaling pathways, including BDNF, and what role synaptic transmission plays in the pathological process of PTSD, drugs were administered to regulate the function of HCN1. ZD7288 is one of the selective HCN channel blockers. After the administration of ZD7288, antidepressant-like effects and improved special learning ability were detected from the results of FST and WMT, suggesting the relief of PTSD symptoms to some extent. The results of chemical assays showed that the administration of ZD7288 inhibited the level of HCN1 and brought significantly increased BDNF protein expression and phosphorylation of mTOR in the PFC and Hip. The analysis of TEM in the PFC showed that the administration of ZD7288 induced an increased thickness of PSD and curvature of synaptic interface and reduced the width of synaptic cleft.

In contrast, 8-Br-cAMP is a cell-permeable cAMP analog that can activate the HCN channel^17^. In our behavioral assays following the delivery of 8-Br-cAMP, more severe anxiety symptoms and weakened spontaneity were detected in the OFT, and the results of the EPMT confirmed anxiety-like behaviors and less exploration in novel environments. In addition, impaired special learning ability was examined by the WTM. It was also found that the delivery of 8-Br-cAMP could activate the HCN1 channel by increasing the expression of HCN1 in the PFC and Hip. It deserves to be mentioned that the rats that underwent the SPS&S procedure showed the impairment behavior in the place navigation and spatial probe tests during the WMT; similar impairments have been reported in numerous studies related to PTSD.

The synaptic transmission or plasticity of the Hip may be critical for spatial learning and memory^40,41^. However, the results of TEM in the present study showed no alteration in the ultrastructure of synapses in the Hip after the delivery of ZD7288 or 8-Br-cAMP. In addition to the Hip, other regions such as the striatum, basal forebrain, and cerebellum, and the interaction among them, also seem implicated in the processes related to learning and memory. Even neuron circuitry may be another important factor^42^. The behavior of rodents in the Morris task involves a complex mechanism in a different brain region; more investigation is needed except for the structure and function of the Hip. In the present study, the administration of ZD7288 reduced the escape latency time in the second and third days in the place navigation test; the escape latency time was lengthened in the second and fourth days after the administration of 8-Br-cAMP although there were no differences in the spatial probe test. These differences illustrate that inhibition and activation of the HCN1 ion channel can improve and aggravate the acquisition of spatial learning of rats in the WMT respectively.

Our previous work indicated that a subanesthetic dose of ketamine could alleviate PTSD-like effects in the rat model induced by SPS&S by inhibiting the expression of HCN1 and up-regulating the BDNF protein levels in the PFC^3^. Results in the present study of the inhibition of HCN1 were consistent with earlier results, but beyond that, the reduced level of HCN1 and higher expression of BDNF were also found in the Hip. Similarly, Kim et al. reported that the silenced HCN1 ion channel in the dorsal Hip had anxiolytic- and antidepressant-like effects, depending on BDNF protein synthesis and activation of the mTOR pathway with an increase in synaptic transmission^8^. There were also early studies describing the possibility of fast changes in synaptic plasticity underlying related diseases such as depression by increasing the phosphorylation and activation of mTOR in the PFC, which leads to a delayed, but sustained induction of synaptic proteins^9^. In addition, perturbation of the mTOR signaling cascade appears to be a common pathophysiological feature of human neurological disorders. However, little is known about the role of mTOR in PTSD. To determine whether mTOR is involved in the development of PTSD, the change of the mTOR signaling pathway was assessed in the present study. We observed a reduced level of pmTOR in the PFC and Hip after the SPS&S procedure that could be reversed by inhibiting of HCN1.

mTOR is normally regarded as a protein kinase involved in translation control and long-lasting synaptic plasticity. pmTOR (the phosphorylated form of mTOR) detected here is an activation of mTOR that is crucial to the cascade of mTOR signaling and functionally linked with local protein synthesis in synapses^14,43^. Studies in cultured cells demonstrate that alpha-amino-3-hydroxy-5-methyl-4-isoxazole propionic acid (AMPA) receptor activation can increase mTOR signaling and synaptogenesis via increased release of BDNF and activation of Akt^44^. Moreover, one study reported that BDNF was an upstream factor controlling mTOR signaling by regulating GluR1 expression during fear memory—one of the core symptoms of PTSD^13^. Based on existing research and our previous study, we analyzed the relationship between BDNF and pmTOR in order to examine the cellular signaling pathways underlying the inhibition of HCN1 in the PFC and Hip. The results of linear regression analysis showed a positive correlation between the levels of BDNF and pmTOR. Subsequently, in order to further elucidate whether BDNF played a key role in activating the mTOR pathway, we carried out mediation analysis. Results showed that BDNF served as a mediator between HCN1 and pmTOR; therefore, it is likely that BDNF is an upstream activator of mTOR, and the inhibition of HCN1 indicates an upregulation of BDNF-mTOR signaling pathways. BDNF and mTOR have been reported to initiate alterations in synaptic plasticity. In the present study, the improved synaptic transmission was examined in the PFC after the inhibition of the HCN1 channel^9,12^; thus, the upregulated BDNF-mTOR signaling pathways may mediate the enhancement of neuron activity and the increase of synaptic transmission in the process of anti-PTSD.

Far less certain, however, is whether the activation of HCN1 by 8-Br-cAMP reduced the positive time significantly in the FST even though more severe anxiety symptoms and weakened exploration ability were detected. A similar study reported that 8-Br-cAMP could reduce the immobility time in the FST in rats, and this effect could be antagonized by co-treatment with selective PKA inhibitor. Evidence from other studies of animal models also suggested that 8-Br-cAMP not only participates in activating the HCN channel but plays an essential role in other pathways accounting for different behaviors and intracellular signal transduction^45,46^. This may be why the activation of HCN1 by the administration of 8-Br-cAMP did not alter the level of pmTOR or the ultrastructure of synapses in the PFC or Hip. In this study, we only detected the lower level of BDNF in the Hip. Another possibility may be that when chronic stress and consequent HCN channel dysfunction desynchronize cortical networks, prefrontal control over behavior, emotion, and cognition could be affected as well^7^. In other words, different physiological functions and abnormal behavior phenotypes might be evoked consistently. This problem deserves further exploration.

It is important to point out that the PFC and Hip as well as other regions in the limbic system appear to play significant roles in PTSD; they should be explored in future investigations. Another question that needs to be investigated is whether synaptic plasticity or transmission is associated with the BDNF–mTOR signaling pathways. In addition to the detection of the ultrastructure of synapses, we intend to examine long-term potentiation and long-term depression—the forms of synaptic plasticity found at excitatory synapses in the mammalian brain—by electrophysiological studies in vivo and to analyze whether they are related to BDNF and pmTOR in the future.

From the data of this study, we found that decreasing the expression of HCN1 in the animal model of PTSD alleviates PTSD-like symptoms, reducing depression-like symptoms and improving spatial learning ability followed by an upregulation of BDNF–mTOR signaling pathways in the PFC and Hip. Meanwhile, the analysis of synaptic ultrastructure showed improved synaptic transmission in the PFC. These data implicated potential effects of a novel ion channel and signaling pathways in PTSD; synaptic translational machinery may also serve a key role. Our research in this area provides a better understanding of the pathophysiology of PTSD and helps lay the foundation for important new insights into molecular targets for treatment.
