# Correction: The potential role of the HCN1 ion channel and BDNF-mTOR signaling pathways and synaptic transmission in the alleviation of PTSD

**DOI:** 10.1038/s41398-020-0795-9

**Published:** 2020-04-14

**Authors:** Lianghui Ni, Yanling Xu, Sixuan Dong, Yujia Kong, Hong Wang, Guohua Lu, Yanyu Wang, Qi Li, Changjiang Li, Zhongde Du, Hongwei Sun, Lin Sun

**Affiliations:** 1 Department of Clinical Medicine, Weifang Medical University, 7166# Baotong West Street, Weifang, Shandong 261053 P. R. China; 2 Department of Psychology, Weifang Medical University, 7166# Baotong West Street, Weifang, Shandong 261053 P. R. China; 3 Department of Psychology, Qingdao University, 16# Qingda First Street, Qingdao, Shandong 266071 P. R. China; 4 School of Public Health and Management, Weifang Medical University, 7166# Baotong West Street, Weifang, Shandong 261053 P. R. China; 5 Department of Neurology, Sunshine Union Hopital, 9000# Yingqian Street, Weifang, Shandong 261000 P. R. China; 6 Department of Psychiatry and Centre for Reproduction Growth and Development, University of Hong Kong, Hong Kong, China

**Keywords:** Depression, Physiology, Molecular neuroscience

Correction to: *Translational Psychiatry*

10.1038/s41398-020-0782-1 published online 20 March 2020

This Article was originally published with errors in Figures 1 and 3.

In Figure 1, “experimental schedule” has been modified to “a”; “a” has been modified to “b”; “b” has been modified to “c” and so on.

In the second line of Figure 3, “g” is misplaced and an additional “h” is written. The errors to both figures have been corrected in the PDF and HTML versions of this Article.

